# Supplementary material for: Onco-miR-21 Promotes Stat3-Dependent Gastric Cancer Progression
Source: Cancers (Basel). 2022 Jan 6;14(2):264. doi: 10.3390/cancers14020264 (PMC8773769; doi:10.3390/cancers14020264)
Supplement: Supplementary file 1 [file cancers-14-00264-s001.zip › cancers-1507488-SI.pdf]

Supplementary Materials: Onco-miR-21 Promotes Stat3-Dependent Gastric Cancer Progression

Janson Tse, Thomas Pierce, Annalisa L. E. Carli, Mariah G. Alorro, Stefan Thiem, Eric G. Marcusson, Matthias Ernst and Michael Buchert

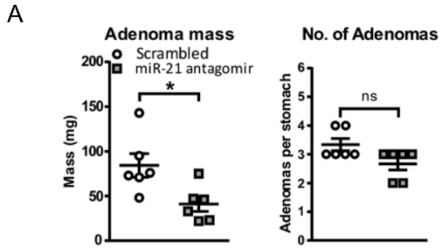

Figure S1. Pharmacological inhibition of miR-21 in vivo attenuates gastric adenoma growth, regardless of mouse strain background. (A) Cumulative tumor mass and number of adenomas from individual Gp130$^{F/F}$ mice (C57Bl/6 inbred background) treated with either scrambled control or miR-21 antagomir. Values shown represent mean ± SEM, with each point representing a single adenoma from n = 3 mice per group.

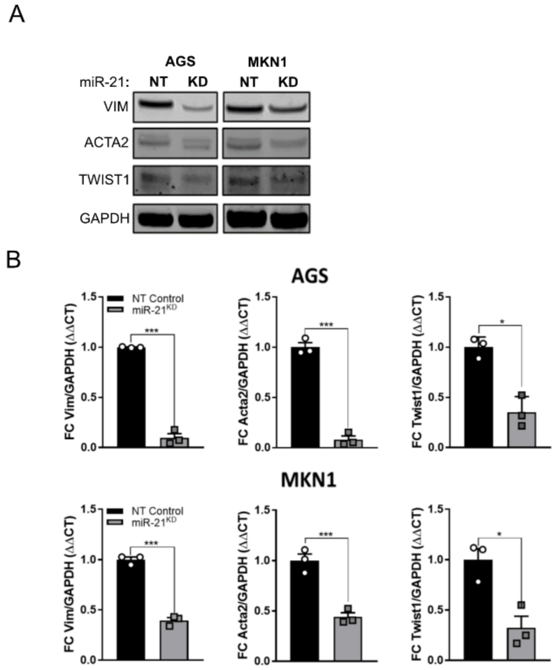

Figure S2. miR-21 regulates the expression of EMT markers in gastric cancer. (**A**) Immunoblot analysis for VIMENTIN (VIM), α-smooth muscle actin (ACTA2) and TWIST1 in AGS$^{miR\text{-}21KD}$ and MKN1$^{miR\text{-}21KD}$ cells compared to non-targeting (NT) control cells. (**B**) Fold change of *VIM*, *ACTA2* and *TWIST1* mRNA expression in AGS$^{miR\text{-}21KD}$ and MKN1$^{miR\text{-}21KD}$ cells compared to NT control cells. Values shown represent mean ± SEM from n=3 experiments. GAPDH was used as a loading control.

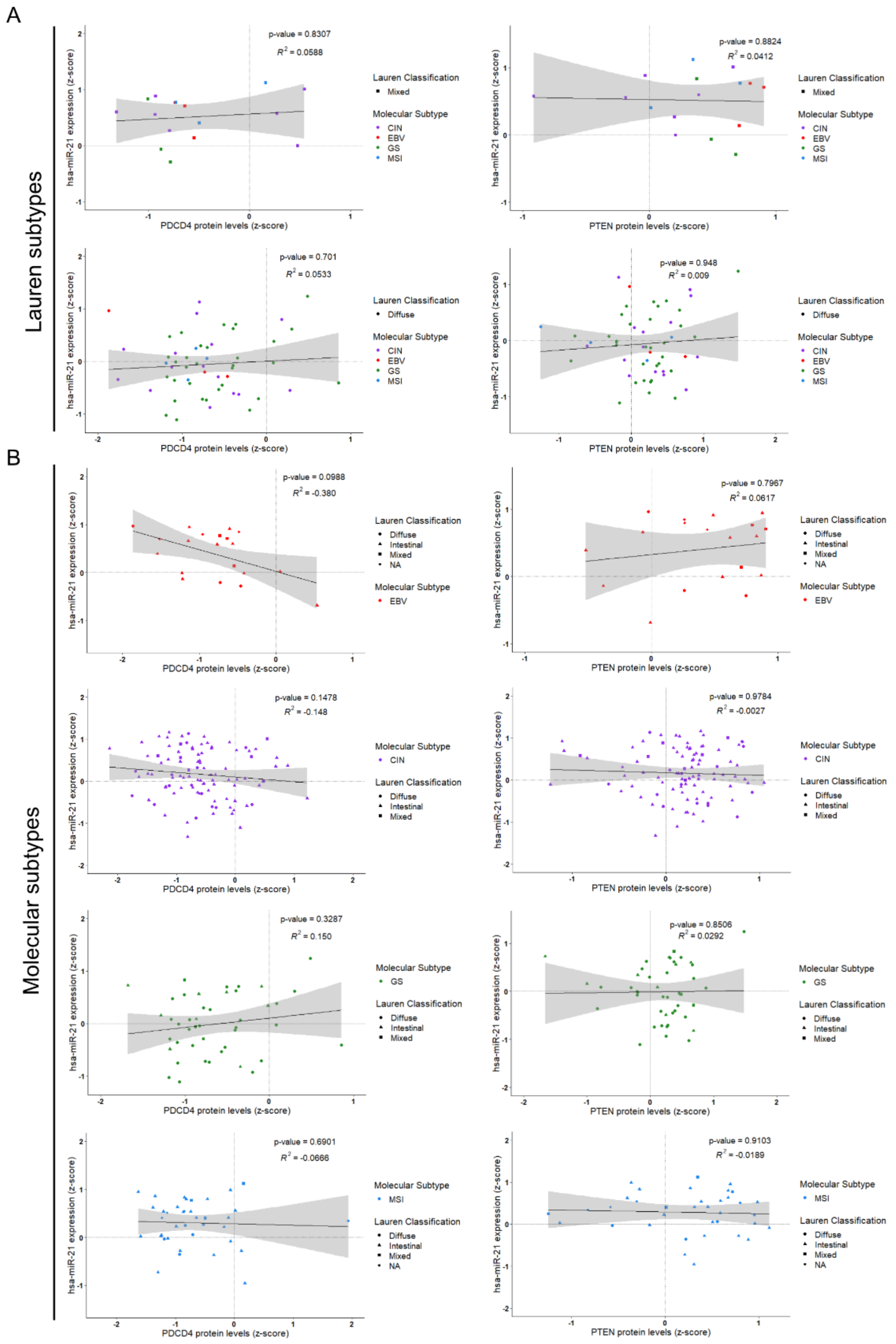

Figure S3. miR-21 correlations with target genes PDCD4 and PTEN in Lauren and Molecular subtypes of GC . **(A)** Correlation analysis of mir-21-5p transcript and mir-21-5p targets PDCD4 and PTEN protein expression levels (z-score, log2 transformed) in the Lauren GC subtypes using the TCGA STAD dataset. **(B)** Correlation analysis of mir-21-5p transcript and mir-21-5p targets PDCD4 and PTEN protein expression levels (z-score, log2 transformed) in the molecular GC subtypes using the TCGA STAD dataset. Each dot represents a single cancer indicating both its molecular subtype and Lauren classification.

Original Western Blots of Figure 1C

pStat3	Gapdh
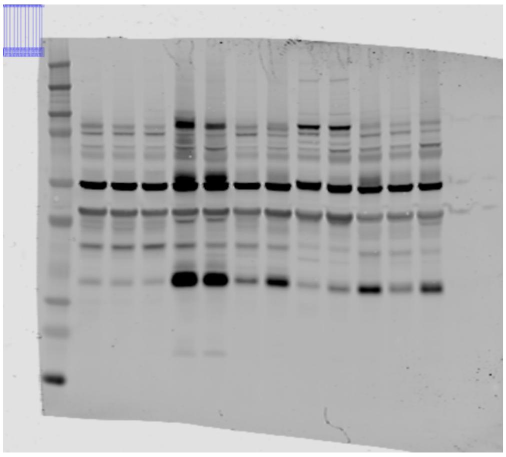	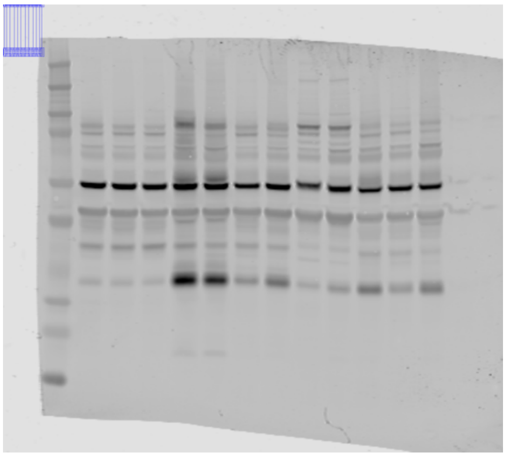

Original Western Blots of Figure 2B

pStat3 Stomach	Gapdh Stomach
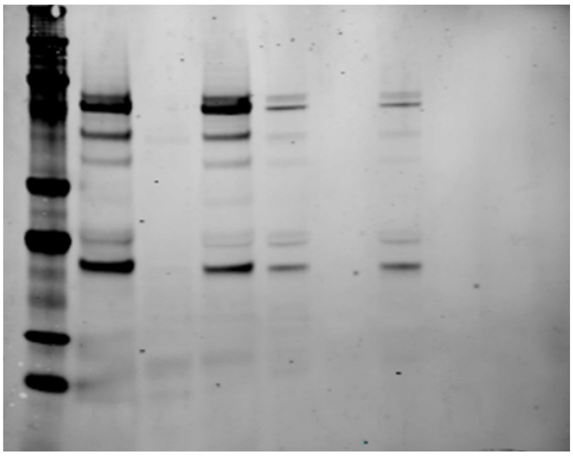	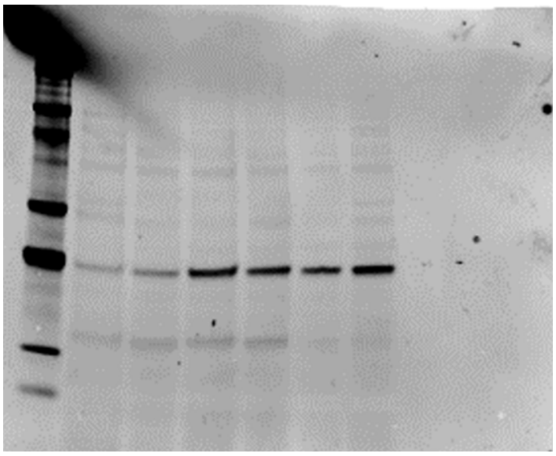
pStat3 Liver	Gapdh Liver
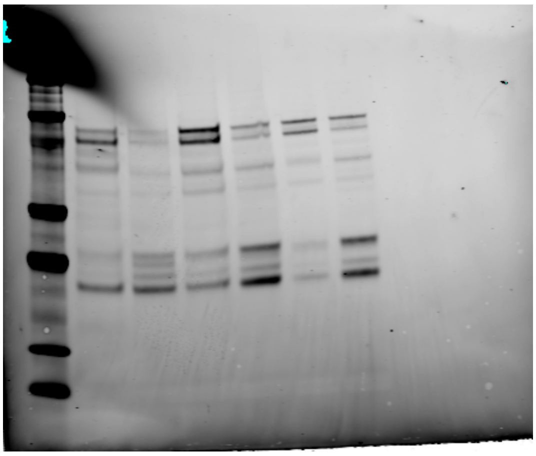	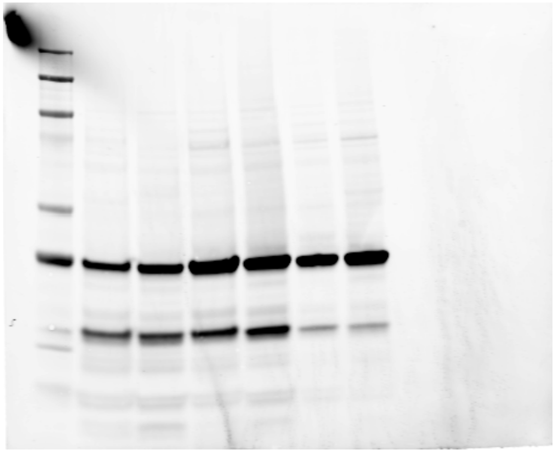

Original Western Blots of Figure 3F

PTEN AGS	PTEN MKN1

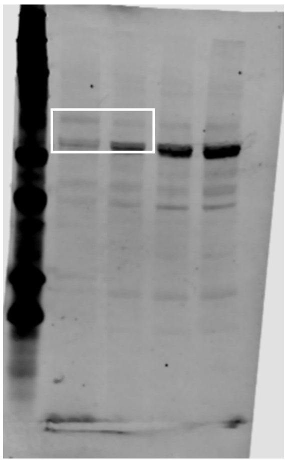	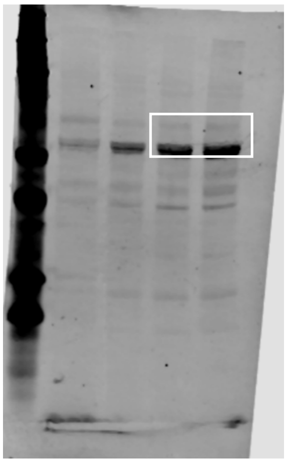
BAX AGS	BAX MKN1
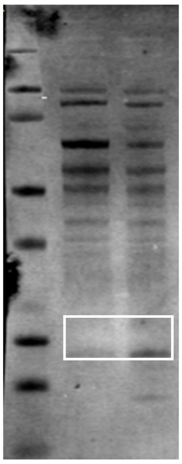	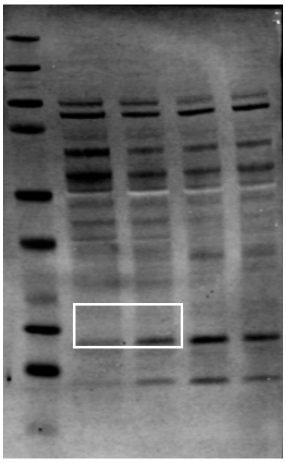
BCL2 AGS	BLC2 MKN1
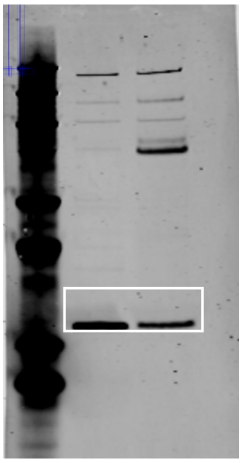	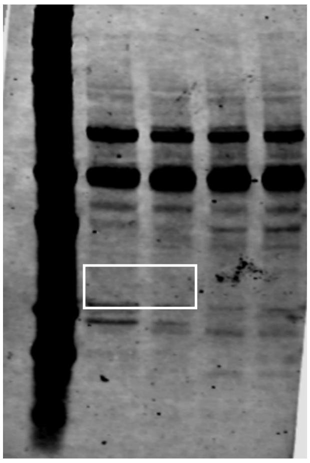
GAPDH AGS	GAPDH MKN1

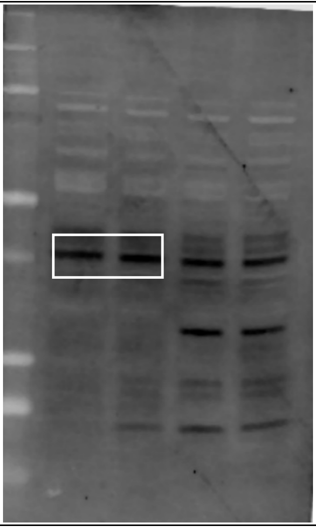	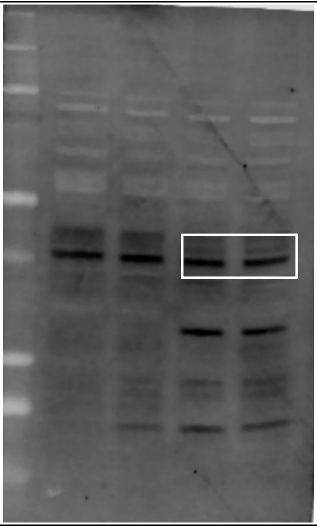
TUBULIN AGS	TUBULIN MKN1
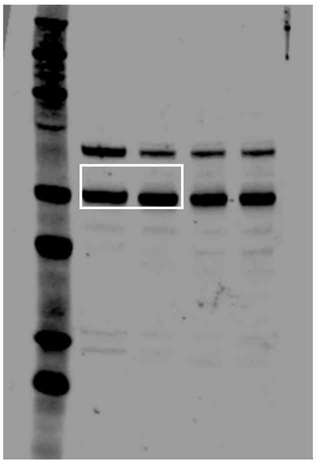	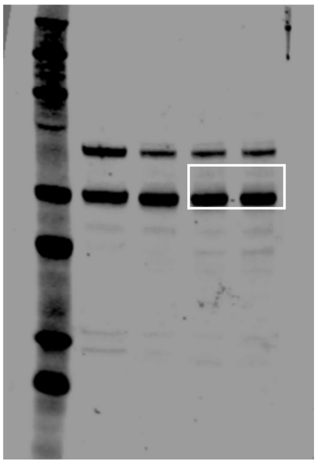

Original Western Blots of Figure S2A

VIM AGS	VIM MKN1
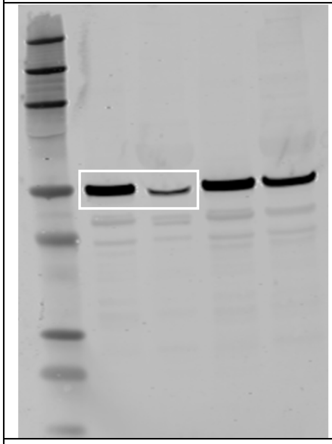	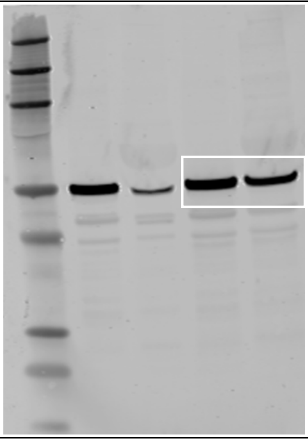
ACTA2 AGS	ACTA2 MKN1

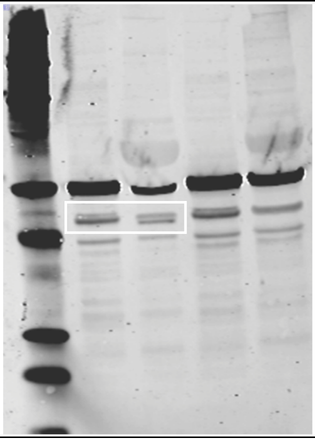	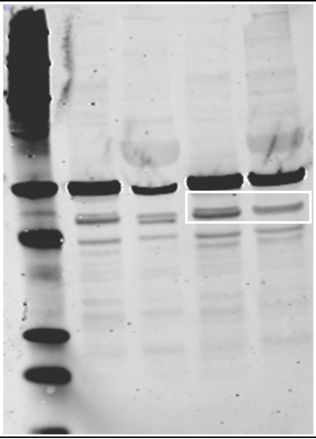
TWIST1 AGS	TWIST1 MKN1
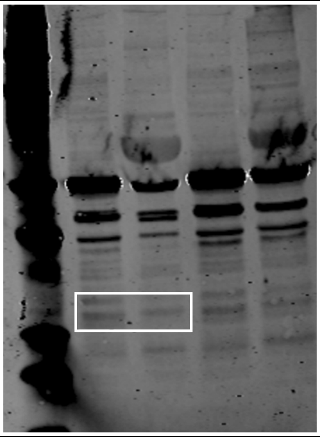	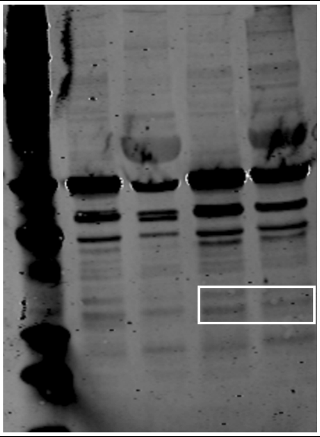
GAPDH AGS	GAPDH MKN1
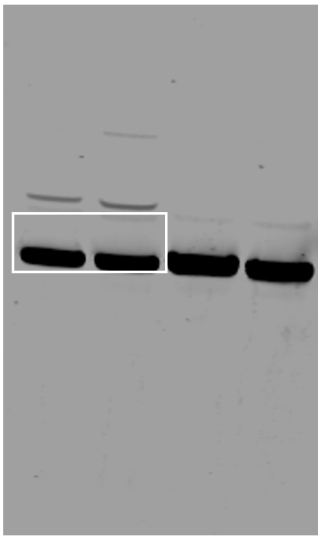	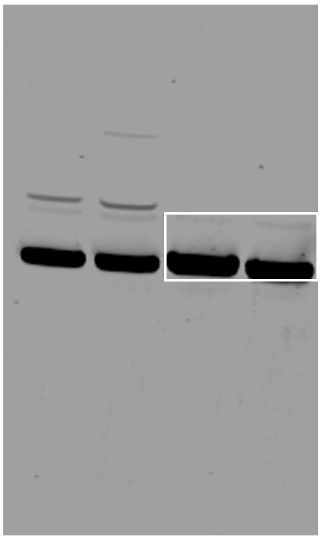

Figure S4. Uncropped western blots.

Table S1. Taqman Q-RT-PCR Primers.

Assay	Accession Number	Sequence (5′-3′)	Species	Assay ID
hsa-miR-21-5p	MI0000077	CAACACCAGUCGAUGGGCUGU	Human	002438
U47	AF141346	TAATGATTCTGCCAAATGAAATATAATGA-TATCACTGTAAAACCGTTCCATTTTGATTCTGAGGT	Human	001223
hsa-miR-21	MI0000077	UAGCUUAUCAGACUGAUGUUGA	Mouse	000397
snoRNA234	AF357329	CTTTTGGAACTGAATCTAAGTGAT-TTAACAAAAATTCGTCACTACCACTGAGA	Mouse	001234

Table S2. Q-RT-PCR Primers.

Primer pair	Sequence (F)orward and (R)everse	Species
VIMENTIN	F: 5′-GTTTCCAAGCCTGACCTCAC<br>R: 5′-GCTTCAACGGCAAAGTTCTC	Human
ACTA2	F: 5′-TCAATGTCCCAGCCATGTAT<br>R: 5′-CAGCACGATGCCAGTTGT	Human
TWIST1	F: 5′-CGGGAGTCCGCAGTCTTA<br>R: 5′-GCTTGAGGGTCTGAATCTTG	Human
GAPDH	F: 5′-GGATTTGGTCGTATTGGG<br>R: 5′-GGAAGATGGTGATGGGATT	Human
Vimentin	F: 5′-GTTGTCTCCTGCGACTTCA<br>R: 5′-GGTGGTCCAGGGTTTCTTA	Mouse
Acta2	F: 5′-GACGTACAACTGGTATTGTG<br>R: 5′-TCAGGATCTTCATGAGGTAG	Mouse
Twist1	F: 5′-GGACAAGCTGAGCAAGATTCA<br>R: 5′-CGGAGAAGGCGTAGCTGAG	Mouse
Gapdh	F: 5′-GTTGTCTCCTGCGACTTCA<br>R: 5′-GGTGGTCCAGGGTTTCTTA	Mouse

Table S3. Antibodies used in Western blot analysis.

Antibody	Company	Cat No.	Species
Phospho-Stat3 (Tyr705)	Cell Signaling	9145	Rabbit
Pten	Cell Signalling	9552	Rabbit
Bcl2	Cell Signaling	2872	Rabbit
Bax	Cell Signaling	2774	Rabbit
Vimentin	Cell Signalling	3932S	Rabbit
Acata2	Abcam	Ab5694	Rabbit
Twist2C1a	Santa Cruz	SC-6269	Mouse
Gapdh	Cell Signaling	2118	Rabbit
IRDye 800 Goat anti-Mouse	LiCor	925-32210	anti-mouse
IRDye 680 Goat anti-Rabbit	LiCor	925-68071	anti-rabbit

Table S4. Antibodies used in immunohistochemistry.

Antibody	Cat No.	Species	Company
Pten	9552	Rabbit	Cell Signalling
Pdcd4	Ab51465	Rabbit	Abcam
Ki67	P6834	Mouse	Sigma
Vimentin	3932S	Rabbit	Cell Signalling
Acta2	Ab5694	Rabbit	Abcam
Twist2c1a	SC-6269	Mouse	Santa Cruz
Phospho-S6	2211	Rabbit	Cell Signalling
Anti-Mouse biotinylated	BA-2001	Mouse	Vector Labs
Anti-Rabbit biotinylated	BA-1000	Mouse	Vector Labs
